# Supplementary figures and images for: Increase of Circulating Monocyte–Platelet Conjugates in Rheumatoid Arthritis Responders to IL-6 Blockage
Source: Int J Mol Sci. 2022 May 20;23(10):5748. doi: 10.3390/ijms23105748 (PMC9144642; doi:10.3390/ijms23105748)

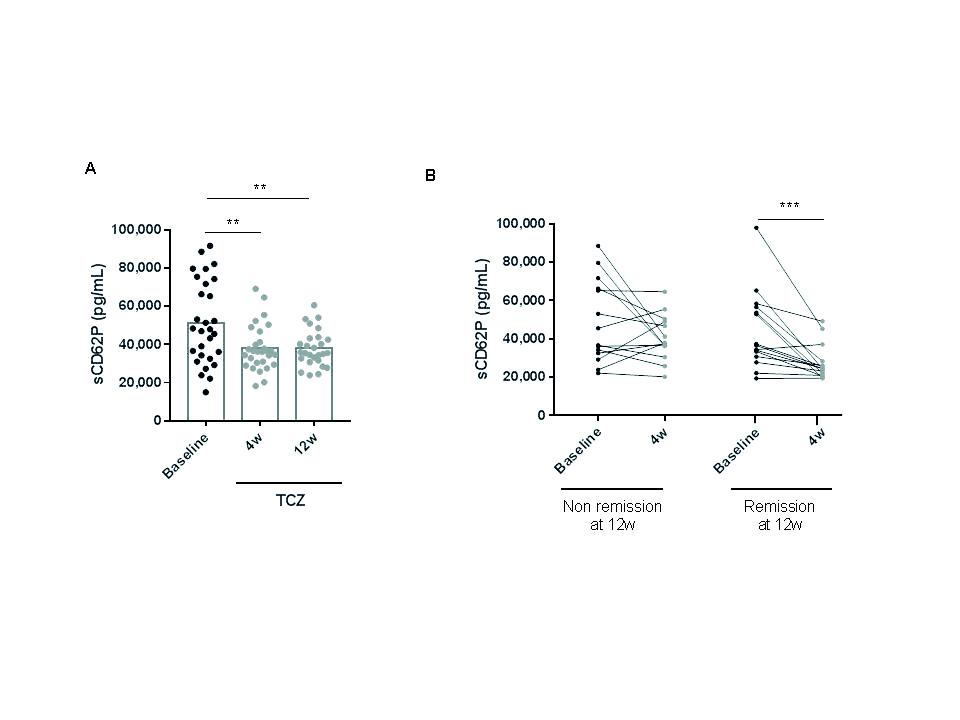

Supplement: Supplementary file 1 [file ijms-23-05748-s001.zip › Figura-S3.jpg]

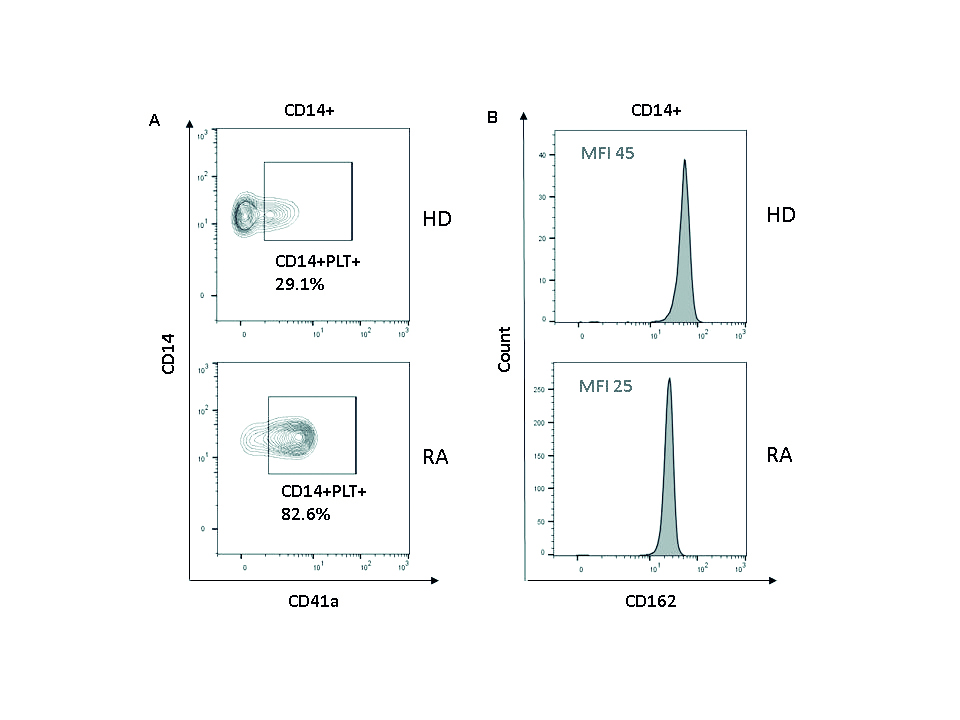

Supplement: Supplementary file 1 [file ijms-23-05748-s001.zip › Figura-S1.jpg]

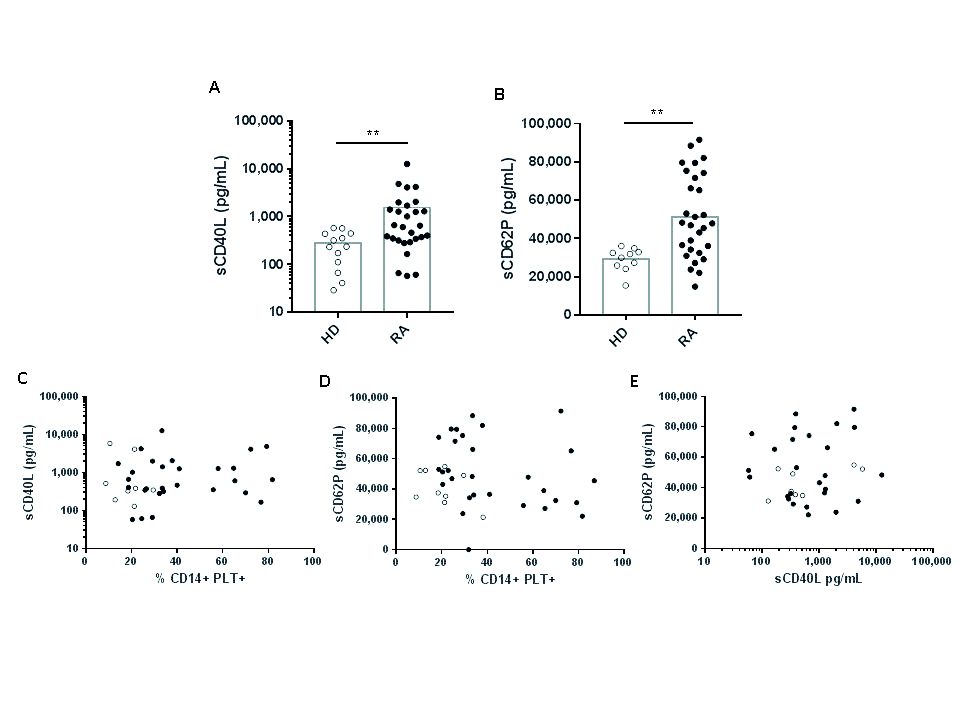

Supplement: Supplementary file 1 [file ijms-23-05748-s001.zip › Figura-S2.jpg]
